# Time and Origin of Cichlid Colonization of the Lower Congo Rapids

**DOI:** 10.1371/journal.pone.0022380

**Published:** 2011-07-20

**Authors:** Julia Schwarzer, Bernhard Misof, Seraphin N. Ifuta, Ulrich K. Schliewen

**Affiliations:** 1 Bavarian State Collection of Zoology, München, Germany; 2 Zoologisches Forschungsmuseum Alexander Koenig, Bonn, Germany; 3 Department of Biology, ISP-GOMBE, Kinshasa-Gombe, Democratic Republic of Congo; University of Lausanne, Switzerland

## Abstract

Most freshwater diversity is arguably located in networks of rivers and streams, but, in contrast to lacustrine systems riverine radiations, are largely understudied. The extensive rapids of the lower Congo River is one of the few river stretches inhabited by a locally endemic cichlid species flock as well as several species pairs, for which we provide evidence that they have radiated *in situ*. We use more that 2,000 AFLP markers as well as multilocus sequence datasets to reconstruct their origin, phylogenetic history, as well as the timing of colonization and speciation of two Lower Congo cichlid genera, *Steatocranus* and *Nanochromis*. Based on a representative taxon sampling and well resolved phylogenetic hypotheses we demonstrate that a high level of riverine diversity originated in the lower Congo within about 5 mya, which is concordant with age estimates for the hydrological origin of the modern lower Congo River. A spatial genetic structure is present in all widely distributed lineages corresponding to a trisection of the lower Congo River into major biogeographic areas, each with locally endemic species assemblages. With the present study, we provide a phylogenetic framework for a complex system that may serve as a link between African riverine cichlid diversity and the megadiverse cichlid radiations of the East African lakes. Beyond this we give for the first time a biologically estimated age for the origin of the lower Congo River rapids, one of the most extreme freshwater habitats on earth.

## Introduction

The rapids of the Lower Congo River rank among the most spectacular habitats for animal life on earth. The Congo basin harbours the highest fish species richness of any river system on the African continent (ca. 700 described species, see also [1]), and especially the lower Congo exhibits a remarkable hydrological, spatial and ichthyological complexity along a short and narrow river stretch [2]–[4]. Before reaching the Atlantic Ocean, all water collected in a drainage basin encompassing one eighth of the African continent (ca. 3.8 mil km^2^) is flushed through an intermittently narrow and deep (up to 200 m) rocky channel, creating the world's most extensive rapids [5]. This approx. 350 km long river section extending from the first rapids near Kinshasa down to the last one upstream from Matadi is geologically young. Its origin is most likely related to a river capture event, i.e. a small coastal river hypothetically tapped the interior Congo basin (sometimes referred to as “Palaeo-lake Congo”), and subsequently created a novel outlet for the whole Congo drainage [5]. Probably before this event, in the Pliocene, the Atlantic Rise had dammed

the course of the Congo River hereby creating a large endorheic basin (“lake”) in the western Congo basin, that nowadays is thought to survive in part as Malebo Pool [5]. Previous assumptions that this lake covered the whole cuvette centrale are unlikely [5]. Age estimates of the river capture are imprecise, varying from early estimates as young as 0.4 mya [6] to 34 mya [7], [8]. Dozens of fish species are endemic to the lower Congo [4] including representatives of the cichlid genera *Steatocranus*, *Nanochromis, Lamprologus*, *Teleogramma* and “*Haplochromis*”. The distribution of all five genera except for the nearly pan-African catch-all genus *“Haplochromis”* is restricted to the Congo basin [9].

In general, cichlids (Perciformes, Cichlidae) are among the most species rich vertebrate groups. Most of their diversity evolved in the great lakes of East Africa, e.g. Lake Malawi, L. Victoria and L. Tanganyika (ca. 1500 species) [10], [11]. Their morphological, behavioural and ecological diversity confined to these single water bodies is considered ideal to study patterns and processes of speciation, which is the reason that African lacustrine cichlid species flocks have been established as evolutionary model systems [10], [12]. In contrast, riverine African cichlid species have been poorly studied. One reason might be that many riverine genera are species poor and exhibit limited morphological diversity, which makes them less attractive study subjects. Up to now the single notable exception is a species complex of southern African rivers, the serranochromines, which may have originally radiated under lacustrine conditions in the now extinct Lake Palaeo-Makgadikgadi [13].

A convincing explanation for the low cichlid species numbers observed in riverine systems is still lacking. One proposed hypothesis is that fluvial systems already inhabited by ecologically diverse fish assemblages generally lack the multiplicity of ecological opportunities necessary for the formation of adaptive radiations [13]. If this is correct, riverine diversification should be best explained by vicariance and geographic isolation and less so by ecological differentiation [13]–[15]. However, the general applicability of this hypothesis remains untested.

**Figure 1:**
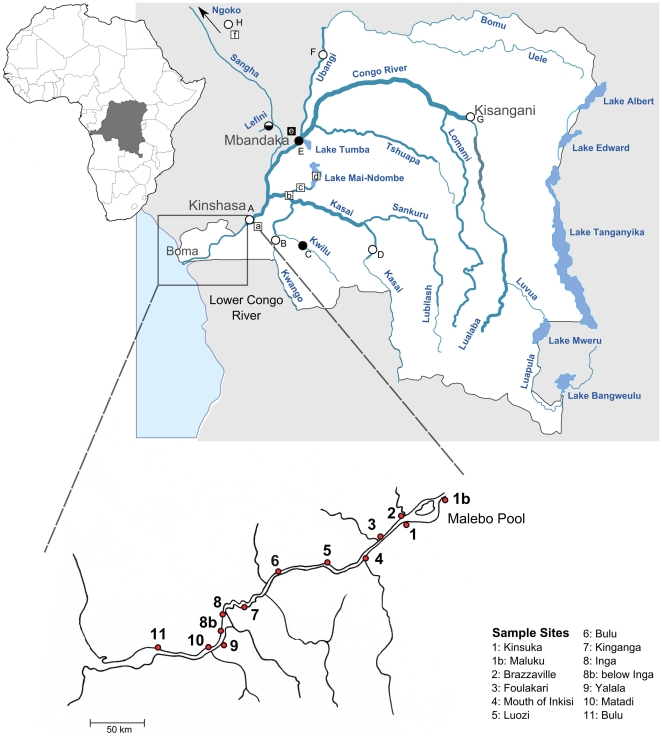
Location map of sampling sites. Circles correspond to Steatocranus and squares to Nanochromis sampling sites. The filled circle and square mark the location of unsamples species. The half filled circle indicate that the species was included in the sequence but not in the AFLP analysis. Letters correspond to non-lower-Congo species distributions: (a) Nanochromis parilus, (b) N. teugelsi, (c) N. nudiceps, (d) N. transvestitus, N. wickleri, (e) N. sp. “Mbandaka”, (f) N. sp. “Ndongo”, (A) *S*. sp. “dwarf”, *S*. sp. “bulky head”, *S. bleheri*, *S*. sp. “Maluku” (B) *S*. sp. “red eye”, (C) S. sp. “Kwilu” (D) *S. rouxi*, (E) *S*. sp. “Mbandaka”, S. sp “Maluku” (F) *S. ubanguiensis,* (G) *S*. sp. “Kisangani” and (H) *S*. sp. “Nki. Sampling locations along the lower Congo are presented in larger scale and marked by red circles. Numbers along the lower Congo correspond to the sample site legend in the lower right side. White background highlights the territory of the Democratic Republic of Congo.

Species of the two lower Congo cichlid genera *Steatocranus* and *Nanochromis* show a scattered distribution of few predominantly allopatric species within the Congo basin (Fig. 1) with a remarkable peak of recently discovered species richness (described and undescribed) endemic to the lower Congo (N (*Steatocranus*)  = 10, N (*Nanochromis*)  = 3). Apart from the lower Congo, *Nanochromis* species are distributed mainly south to the central Congo basin in Lakes Tumba and Mai Ndombe and adjacent rivers (*N. wickleri* and *N. transvestitus*, *N. nudiceps*) or in the Kasai River drainage (*N. teugelsi*). One undescribed species (*N.* sp. “Ndongo”) has recently been discovered in rivers Ngoko and Sangha (pers. obs.), both forming a northwestern Congo tributary, and another yet undescribed species (*N*. sp. “Mbandaka”) is known from the Congo mainstream around Mbandaka [16]. *Nanochromis parilus* is distributed in the lower Congo but also above Malebo Pool e.g. at Maluku. *Steatocranus* species occurring outside the lower Congo are distributed either in northern tributaries (*S*. sp. “Nki”, *S. ubanguiensis* and *S*. sp. “Lefini” from Ngoko, Ubangi and Lefini rivers) or south to the Congo mainstream (*S. rouxi*, *S*. sp. “red eye”, S. sp. “Kwilu” from Kasai, Kwango and Kwilu rivers), or in the Congo proper (*S*. sp. “dwarf”, *S*. sp. “bulky head”, *S. bleheri*, *S*. sp. “Maluku”, *S*. sp. “Mbandaka” and *S*. sp. “Kisangani” from around Malebo Pool, Maluku, Mbandaka and Kisangani). The haplotilapiine genus *Steatocranus* consists of rheophilic species whereas within the chromidotilapiine genus *Nanochromis* adaptations to high current are less obvious [4], [17], [18]. Habitat preferences differ between the mainly rock-dwelling *Steatocranus* and the more sand-dwelling *Nanochromis* (pers. obs.). Lower Congo *Steatocranus* are characterized by divergence in trophic traits indicating ecologically differentiated trait utility, i.e. dentition used for algae scraping (“aufwuchs feeding”), molluscivory and drift feeders ([4], pers. obs.). Recent surveys by different teams along multiple locations along the Lower Congo discovered that the species distribution of cichlids along the Lower Congo is not homogeneous. Both *Nanochromis* and *Steatocranus* species can be confined to short rapids stretches, and partially occur syntopically with close congenerics, whereas other species are less restricted in their distribution and/or represent the single genus representative in a selected rapids stretch.

Greenwood [19] has defined “species flocks” as systems in which multiple species have diversified from a single common ancestor in a geologically restricted area. This definition is assessed by three diagnostic criteria characterising a fish species flock: (1) a geographical circumscription, (2) a high level of endemism and (3) a close phylogenetic relationship (e.g. Salzburger & Meyer [20]). Following this definition, the lower Congo River species assemblages of the genera *Steatocranus* and *Nanochromis* should each qualify as riverine cichlid “species flocks”.

Based on extensive AFLP and DNA sequence data and an almost complete taxon sampling, we use a phylogenetic approach to decipher age, origin and pattern of local diversification of these two distantly related lower Congo cichlid genera. We provide for the first time age estimates for the colonization of the lower Congo rapids, which also serve as the minimum age of their geological formation.

## Materials and Methods

### Sampling

Samples from the lower Congo River were collected during low water season (June – August) between 2005 and 2008 in the Democratic Republic of Congo and in 2004 in the Republic of Congo. Our sampling focussed on sequentially arranged regions along the lower Congo River (Fig. 1). In addition, almost all known *Steatocranus* and *Nanochromis* species from surrounding rivers were included in the analyses (Table S1). Based on the phylogenetic analysis of Schwarzer et al. [18], the haplotilapiine cichlids *Tilapia busumana* (West Africa), *Tilapia cf. bilineata* (Central Congo basin), *Eretmodus cyanostictus* (L. Tanganyika) and *Lamprologus mocquardi* (Central Congo Basin) were chosen as outgroups for the *Steatocranus* dataset, and the Congolian chromidotilapiine genera *Teleogramma* and *Congochromis* [17] served as outgroups for the *Nanochromis* dataset.

### Molecular methods

The mitochondrial gene ND2 was amplified using primers ND2Met and ND2Asn and sequenced using primers ND2Met and ND2Trp [21]. ND2 datasets consisted of 1029bp for *Steatocranus* (N = 133) and 980 bp for *Nanochromis* (N = 78). Additionally the mitochondrial 16S, the first nuclear intron of S7 [22] and the ribosomal genes ENC1, Ptr and SH3PX3 [23] were sequenced for selected *Nanochromis* and *Steatocranus* species (Table S2) in order to incorporate the data in a larger cichlid phylogenetic framework from Schwarzer et al. [18]. Amplifications were performed in 10 µl volumes containing 5 µl Multiplex Mix (Qiagen), genomic DNA 1 µl, 0.8 µl of each Primer (2,5 nmol), Q-Solution (Qiagen) and water. Amplifications of all fragments were carried out in 40 cycles according to the temperature profile: 15 min at 95°C (initial denaturation), 30 s at 95°C, 30 s at 60°C, 90 s at 72°C, and finally 10 min at 72°C. PCR products were purified with ExoSAP-IT (USB) and diluted with 10 µl - 20 µl HPLC water, depending on product concentration. Sequencing was performed according to standard methods, using Big Dye 3.1. (Applied Biosystems). DNA sequences were read using an ABI 3130xl DNA sequencer (Applied Biosystems). Chromatograms were assembled using SeqMan v. 4.03 included in the Lasergene software package (DNASTAR) and manually proof read. Alignments were conducted using the Clustal W algorithm implemented in BioEdit v. 7.0.4.1. The multilocus dataset (N = 65) of all sequenced markers resulted in a data matrix of in total 4108 bp comprised of S7 (first intron): 528 bp, 16SrRNA: 513 bp, ND2: 993 bp, ENCI: 707 bp, Ptr: 688 bp and SH3PX3: 679 bp. A modified protocol of the original AFLP method [24] as suggested in Herder et al. [25] was used. The following twenty *EcoRI/MseI* primer pairs with three selective bases were used for selective AFLP amplification: ACA*-CAA; AGG*-CTG; ACC*-CTA; ACT*-CAT; ACA*-CTT; AGG*-CAC; AGC*-CAG; ACT*-CTC; ACC*-CAC; AGG*-CTA; AGC*-CTT; ACT*-CAA; ACA*-CAC; AGG*-CAG; ACC*-CTG; ACT*-CTG; AGC*-CAT; AGG*-CTT; ACA*-CAG; ACT*-CAC. Bands were visualized on an AB 3130 sequencer (Applied Biosystems) and Genemapper® v 4.0. software using the size standard ROX 500 XL. Peaks between 100 and 499 bases could be scored unambiguously for presence/absence. The analysis was conducted automatically using Genemapper® v 4.0. Eight to eleven individuals were genotyped two (N = 8) to three times (N = 3) to test for reproducibility. Considering the standard error of automated sequencers, pairs of neighbouring bins whose minimum distance between each other was less than 0.25 bp and also bins containing fragments differing more than 0.65 bp in size were removed from the dataset. In the *Steatocranus* dataset samples were run in two batches. Therefore bins with fragments that differed by more than 20% relative frequency between the two runs were removed. This step in primary data acquisition decreases rather than increases the likelihood of detection of population structure and was chosen to prune the data set from plate specific effects. The error rate per individual was calculated as the ratio between observed number of differences and the total number of scored fragments [26]. The mean error rate for the *Steatocranus* and *Nanochromis* AFLP datasets was 3% and 2% respectively.

### Phylogenetic inference

Alignment of the sequences was conducted using BioEdit (ClustalW), followed by a masking of ambiguous alignment positions using ALISCORE v.0.2 under default settings [27]. A ML analysis was conducted for all datasets with RAxML v. 7.0.3 [28] using the GTR+Γ model and the rapid bootstrap algorithm with a subsequent search for the best-scoring ML tree. Branch support was evaluated with 1000 non-parametric bootstrap (BS) pseudo-replicates. Model parameters were estimated separately for each possible partition (genes and codon positions separately). For BI, best-fitting models of sequence evolution were estimated using the Bayes Factor Test [29]. Bayesian analyses were performed using MrBayes 3.1.2 [30] with 20^6^ generations starting with random trees and sampling of trees every 500 generations. To ensure convergence the first 20^5^ generations were treated as burn-in and excluded. The remaining trees from all Bayesian analyses were used to build a 50% majority rule consensus tree. For the AFLP data a neighbour-joining tree based on Link et al. [31] distances was calculated using TREECON 1.3b [32]. Bootstrap values were calculated based on 1000 pseudoreplicates.

### Dating and diversification rates

Divergence times of *Steatocranus* and *Nanochromis* were estimated using a relaxed-clock Bayesian approach implemented in BEAST v. 1.5.3 [33]. To set calibration points, *Nanochromis* and *Steatocranus* sequences were integrated in an already published dataset [18] based on multiple genes. The ML tree was used as starting tree. The Yule model was selected as tree prior and an uncorrelated lognormal model was used to estimate rate variation along branches. The same priors used in Schwarzer et al. [18] were applied: an exponential prior (zero offset 5.98 mya) at the root of all oreochromines [34] and a uniform prior (53–84 mya) at the root of all African cichlids except *Heterochromis* [35]. For a detailed discussion on the choice of priors see Schwarzer et al. [18]. The analysis was run 30^6^ generations and the effective sample size was checked using Tracer v. 1. 4. [36].

## Results

### Molecular phylogenetics

AFLPs provided an almost fully resolved phylogenetic tree for both lower Congo cichlid genera. In the *Steatocranus* dataset one primer combination (ACT*-CTC) was excluded from further analysis as all samples showed off-scale peaks. The final AFLP datasets were composed of 145 (141 *Steatocranus,* 4 outgroup taxa) and 76 (70 *Nanochromis*, 6 outgroup taxa) specimens with 3031 (*Steatocranus*) and 3658 AFLP loci (*Nanochromis*) respectively. Of these 2101 (1706 without outgroups) fragments of the *Steatocranus* and 2182 (1566 without outgroups) of the *Nanochromis* AFLP dataset were polymorphic. The ND2 alignments had 293 (*Nanochromis*) and 285 (*Steatocranus*) variable sites and empirical base frequencies of A = 0.26, C = 0.35, G = 0.11, T = 0.28 and A = 0.26, C = 0.35, G = 0.11, T = 0.28, respectively. The concatenated multigene dataset consisted of two mitochondrial (ND2 and 16S) and four nuclear loci (ENC1, Ptr, SH3PX3 and S7) for 65 selected taxa (Table S2). 347 bp were excluded from further analyses due to alignment ambiguities within the S7 intron (16 positions) and due to saturation in the 3rd codon position of the mitochondrial ND2 locus. The final alignment had 3761 bp. Based on the Bayes factor test, we used a partition separating Exons, 16S and ND2 (first and second codon position) and the S7 intron. The HKY model resulted as best fitting model for all partitions except for nuclear exons (ENC1, Ptr and SH3PX3), which were assigned to GTR +Γ. The dataset had 605 variable sites and empirical base frequencies of A = 0.26, C = 0.26, G = 0.23, T = 0.25.

### Intragroup phylogenetic patterns

**Figure 2:**
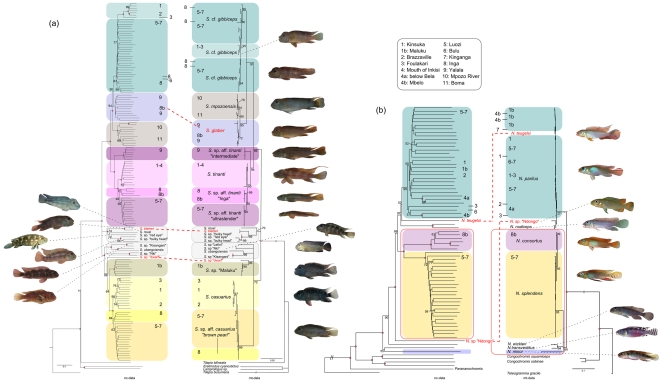
Phylogenetic trees based on nc- and mt-datasets. The datasets comprises mitochondrial sequences of ND2 and over 2000 AFLP markers for (a) Steatocranus and (b) Nanochromis. Black numbers at nodes refer to bootstrap-values (BS, 1000 pseudo-replicates) of the ML run (right side) or the neighbour joining tree (left side). Filled circles represent a 100% BS support. Major groups within the phylogeny are marked with coloured frames. Species that are placed differently in AFLP and mt-trees are marked in red. The red frames in *Nanochromis* trees indicate differences in sistergroup-ralationships.

Phylogenetic analyses of the AFLP and the mtDNA dataset based on ND2 yielded mostly congruent results concerning intrageneric relationships (Fig. 2). The lower Congo species were polyphyletic with respect to central and upper Congo taxa, since in both cases two distantly related lower Congo lineages were present.

In *Nanochromis* one phylogenetic group is composed of the central and downstream lower Congo endemics *N. consortus* and *N. splendens* (BS in nc- and mt-phylogenies 100 and 95 respectively) and a second of *N. parilus* (from the upper and central lower Congo and Maluku), *N. nudiceps* (from a river close to Lake Mai Ndombe) and *N. teugelsi* (from the lower Kasai, BS = 97 and  = 77, Fig. 2). *Nanochromis minor* from the central lower Congo sampling location Kinganga and *N. transvestitus* from Lake Mai Ndombe appears as sistergroup to all other *Nanochromis* in the AFLP dataset (BS = 100) but as sistergroup to the *N. splendens*/*N. consortus* group in the mtDNA dataset (including *N. wickleri*, BS = 69). The phylogenetic positions of *N.* sp “Ndongo” from the Sangha drainage and *N. teugelsi* from the Kasai also remain unresolved. *Nanochromis* sp “Ndongo” either clusters with *N. parilus/N. nudiceps/N. teugelsi* in the mtDNA dataset (BS = 77) or appears as sistergroup to all *Nanochromis* except for *N. minor* and the central Congo species *N. wickleri* and *N. transvestitus* in the AFLP tree (BS = 91). *Nanochromis teugelsi* from the Lower Kasai, a southern tributary to the Congo, is part of the *N. parilus* clade in the mtDNA dataset (BS = 77) but appears as weakly supported (BS = 52) sistergroup to either *N. parilus* or *N. nudiceps* (BS = 46) in the AFLP dataset.

*Steatocranus* splits into three major monophyletic groups. One is composed of the lower Congo endemics *S. casuarius* and *S.* sp. aff. *casuarius* “brown pearl”, *Steatocranus* sp. “Maluku” from upstream of Pool Malebo, *S*. sp. “dwarf” and species from the northern tributaries Ubangi and Ngoko and from the upper Congo near Kisangani (*S*. sp. “Kisangani”, *S. ubanguiensis*, *S*. sp. “Nki” and based on the mt- tree also *S*. sp. “Lefini”, Fig. 2). *Steatocranus* sp. “Maluku” appears as sistergroup to the *S.* cf. *casuarius* clade (BS = 64) in the nc- but to *S*. sp. “dwarf” in the mt- dataset (BS = 96). The second major group is composed of the lower Congo endemics *S.* cf. *gibbiceps, S. mpozoensis*, *S. glaber* and four distinct *S.* cf. *tinanti* clades (BS = 100 in the nc and BS = 91 in the mt-dataset, Fig. 2). The relationship of *S. glaber* is ambiguous as it is sister to *S. mpozoensis* in the mt- dataset (BS = 95) but to *S. gibbiceps* in the AFLP dataset (BS = 90). *Steatocranus* species occurring outside of the Lower Congo roughly form two phylogenetic clusters according to their major geographic distribution either north (“North” clade, Congo mainstream/Ubanghi/Sangha/Lefini) or south (“South” clade, Kasai/Kwango and Pool Malebo) to the Congo mainstream. The third major group, composed of species from the southern Congo tributaries and from the Congo mainstream (*S. rouxi*, *S.* sp. “red eye”, *S.* sp. “bulky head”, and *S. bleheri*), appears polyphyletic in all datasets. In 1000 bootstrap replicates they appear in almost equal measure as sistergroup to all remaining *Steatocranus* (BS = 47, Fig. S1), or alternatively, to the lower Congo *S.* cf. *gibbiceps, S. glaber, S. mpozoensis* and *S.* cf. *tinanti* (BS = 44, Fig. S1). Within this monophyletic group, *S. rouxi* from the upper Kasai River appears as sistergroup to the remaining taxa from southern tributaries in the mt- but to *S*. sp. “red eye” and *S*. sp. “bulky head” in the nc- phylogeny (Fig. 2).

### Dating and diversification rates

**Figure 3:**
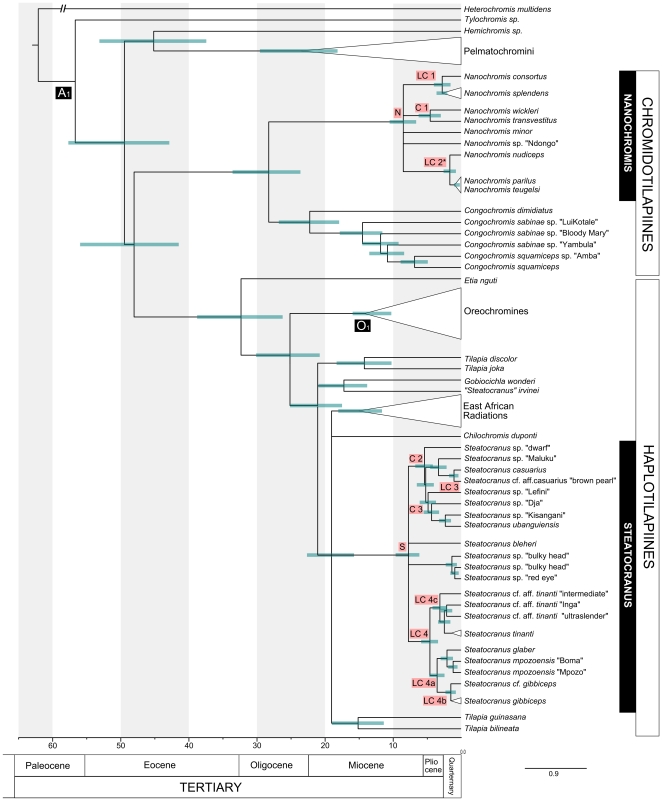
Chronogram showing divergence time estimates for Steatocranus and *Nanochromis.* The chronogram was calculated based on the ML tree. A partitioned Bayesian analysis implemented in BEAST was used to estimate divergence times. Time constraints were used following Schwarzer et al. [18]: A_1_ 53–84 mya (uniform prior), published age estimate based on non-cichlid fossils [35] and O_1_ 5.98 mya (lower bound), the age estimate for *Oreochromis lorenzoi*† [34]. The chronogram shows 95% credibility intervals (HPC, green bars). For nodes marked with letters, age estimates (95% HPC and mean heights) are given in Table 1. The asterisk marks the non-endemic lower Congo clade including *N. parilus* and *N. teugelsi*. *Nanochromis parilus* is distributed in the lower Congo but can also be found at Maluku upstream of Malebo pool. For simplification clear monophyletic groups were combined and shown as triangles.

The age of the most recent common ancestor (MRCA) of *Nanochromis* is estimated at 8.36 (6.5 - 10.4) mya (Fig. 3, Node N). Median ages for clades containing lower Congo taxa (Node LC 1: *N. splendens* and *N. consortus,* Node LC 2*: *N. parilus*, *N. teugelsi* and *N. nudiceps*) are estimated at 2.67 (1.5–3.9) mya and 1.6 (0.7–2.5) mya. The two lacustrine species from the central Congo, *Nanochromis wickleri* and *N. transvestitus* (Node C1), diverged 4.4 (3–6.2) mya based on our data (Fig. 3, Table 1).

**Table 1:** Age estimates.

Node	Date estimates in Myr					
	Baysian Inference (95% credibility intervals)					
	**This study**		**Schwarzer et al. ** [**18**]		**Genner et al. ** [**55**]	
**A_1_**	**55.5**	(42.8, 57.6)	**56.7**	(53.0, 64.2)	63.7 (N)	(46.6, 79.6)
**O_1_**	**12.8**	(10.2, 15.9)	**12.8**	(8.9, 16.8)		
N	**8.36**	(6.5, 10.4)				
S	**7.7**	(6.1, 9.6)	**10.7**	(7.4, 14.1)		
LC1	**2.7**	(1.5, 3.9)				
LC2*	**1.6**	(0.7, 2.5)				
LC3	**0.9**	(0.3, 1.7)				
LC4	**4.5**	(3.3, 5.8)	5.7**	(3.6, 8.37)		
LC4a	**3.4**	(2.4, 4.6)	3.1**	(1.43, 5.14)		
LC4b	**1.4**	(0.7, 2.3)				
LC4c	**3.0**	(4.5, 7.6)				
C1	**4.4**	(3.0, 6.2)				
C2	**5.3**	(4.1, 6.7)	6.9***	(4.1, 10.17)		
C3	**4.8**	(3.2, 5.5)				

Priors A_1_ and O _1_ were taken from Schwarzer et al. [18] and resulting age estimates were compared with publishes studies [18], [55] when possible. LC2* contains Nanochromis parilus and N. teugelsi, which are not endemic to the lower Congo. Two or three asterisks (** or ***) mark nodes whose node ages were estimated either without S. mpozoensis (**) or S. sp. “Maluku” (***) and are thus not one to one equivalent to nodes LC 4, LC 4a and C2 in this study. Age estimates given for node A_1_ from Genner et al. [55] correspond to their dataset calculated with Gondwana priors.

The age of the MRCA of the genus *Steatocranus* is estimated at 7.7 (6.1–9.6) mya (Fig. 3, Node S). For the lower Congo *Steatocranus* clades age estimates were as follows: node LC 3: *S. casuarius* species complex 0.94 (0.3–1.7) mya, node LC 4: *S.* cf. *gibbiceps*, *S. glaber*, *S. mpozoensis* and *S.* cf. *tinanti* 4.48 (3.3–5.8) mya, node LC 4a: *S.* cf. *gibbiceps*, *S. glaber* and *S. mpozoensis* 3.43 (2.4–4.6) mya, node LC 4b: *S. gibbiceps* species complex 1.42 (0.7–2.3) mya, node LC 4c: *S. tinanti* species complex 3.03 (4.5–7.6) mya (Fig. 3). The age for MRCA of the clade containing *S*. sp “dwarf”, *S*. sp. “Maluku”, *S. casuarius*, *S*. sp. aff. *casuarius* “brown pearl” and species from the northern tributaries (Node C2) was estimated at 5.3 (4.1–6.7) mya. The MRCA of the species from the northern tributaries alone was estimated at 4.8 (3.2–5.5) mya (Fig. 3, Node C3). The estimation of dates for the origin of species from surrounding lakes and rivers is problematic as ambiguous signal masked the phylogenetic relationships at the base of the lower Congo clades (see section above).

## Discussion

### Potential colonization of the lower Congo rapids

Phylogenetic inferences based on a fully representative taxon sampling revealed that the ancestors of lower Congo species of both studied genera reached the rapids in at least two allochronic events and then further differentiated within the lower Congo. Though the age estimate for the MRCA of *Steatocranus,* at 7.7 (6.1–9.6) mya, is slightly younger than in the previous study by Schwarzer et al. (7.4–14.1 mya), [18], the 95% confidence intervals still highly overlap and are on average smaller (Table 1). The inclusion of more terminal taxa combined with a different gene composition used for the analysis might have caused the differences. Age estimates, however, should in the present context not be seen as fixed numbers but as rough chronological placements of evolutionary processes.

**Figure 4:**
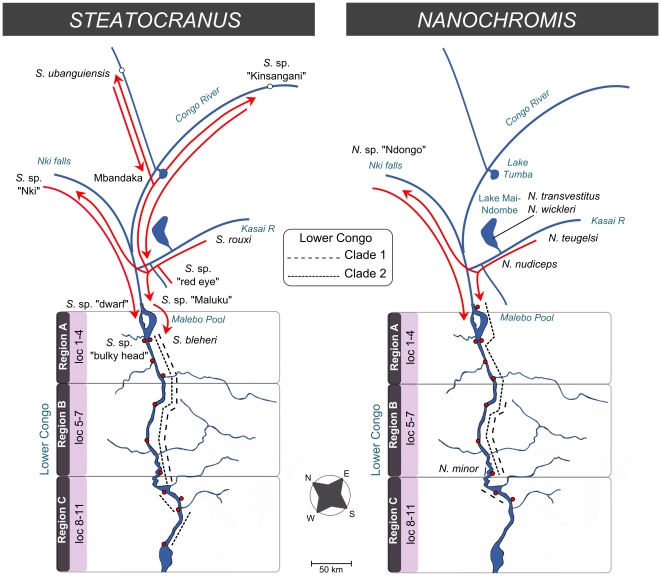
Potential colonization scenarios for *Steatocranus* and *Nanochromis*. Potential colonization scenarios are shown separately for both genera. The red arrows indicate potential dispersal routes of Steatocranus and Nanochromis precursors. Non-lower Congo taxa are written in black. Current distribution ranges of the major lower Congo phylogenetic clades (see Fig. 2) are represented by different kinds of dotted lines (see figure legend). Clade 1 (Steatocranus): S. cf. casuarius species pair, clade 2 (Steatocranus): *S*. cf. *tinanti*/ *S*. cf. *gibbiceps*/ *S. glaber* and *S. mpozoensis*, clade1 (Nanochromis): N. splendens and N. consortus, and clade 2 (Nanochromis): N. parilus. The trisection of the lower Congo is shown in combination with sampling sites (indicated by red dots) along the lower Congo. The upper part of the Congo River is presented highly simplified for a better understanding.

Apparent cyto-nuclear tree incongruence hampers the reconstruction of some colonization patterns in both genera (Fig. 2). In *Steatocranus* a well supported relationship is identified between the “North” clade species and the lower Congo *S.* cf. *casuarius* clade (including *S*. sp. “Maluku” and *S*. sp. “dwarf”), indicating that the ancestors of present day *S.* cf. *casuarius* colonized the lower Congo (at about 3.23 mya) from Northern populations distributed presently in the upper Congo near Kisangani, downstream of Mbandaka and in northern Congo tributaries (Ubangi and Sangha Rivers). The phylogenetic signal concerning the position of the Southern populations is ambiguous. Two alternative topologies are most frequently found among the bootstrap replicates (Fig. S1), supporting two slightly different colonization scenarios with regard to the lower Congo: In the first scenario, a simultaneous dispersal of southern precursors into the lower Congo as well as into the northern tributaries took place, i.e. seeding both the present day *S*. cf. *tinanti*/*S. mpozoensis*/*S. glaber*/*S*. cf. *gibbiceps* clade (ca.4.48 mya, Fig. 3) and the northern clade (BS = 47, Fig. S1). Subsequently a secondary colonization wave from northern tributary species and from the Congo mainstream then founded the younger lower Congo *Steatocranus* cf. *casuarius* clade (ca. 3.23 mya, Fig. 3). In the second scenario, an early vicariance event separated already existing southern and northern tributary populations which then founded the *S*. cf. *tinanti*/*S. mpozoensis*/*S. glaber*/*S*. cf. *gibbiceps* clade (BS = 44, Fig. S1) from the South and later the *Steatocranus* cf. *casuarius* clade from the North (Fig. 4).

In *Nanochromis* the nc-dataset indicates an initial colonization from the lineage comprising central lacustrine species *N. transvestitus, N. wickleri* and the lower Congo species *N. minor*. This scenario resembles that of *Steatocranus*, with older southern populations seeding the lower Congo (mt-signal, Fig. 2) and (potentially) the Northern tributaries (nc- signal, Fig. 2) followed by a second colonization wave from central Congo (ca.1.6 mya) and potentially from the North (*N*. sp. “Ndongo”, mt-signal, Fig. 2).

Both younger phylogenetic clades (*S*. cf. *casuarius* and *N. parilus*) exhibit a distribution limited to the upstream and central lower Congo. This parallel pattern implying two colonization waves into the lower Congo in both genera (Fig. 4) might indicate that geological factors have shaped the evolution of these distantly related genera in parallel. One palaeogeological event that may have played a part was the gradual coastal uplift of the West African continental margin (“Atlantic Rise”) since the early Miocene [37] which potentially went along with changes in drainage pattern of the greater lower Congo region [38].

### The geological age of the lower Congo River drainage

Analyses of fluvial sediments in the Gulf of Guinea show that major offshore deposits were not present in the region of the present day Congo mouth until relatively recently, but that major parts of central Africa's interior drainage discharged in the Cretaceous and Oligocene (65 to 36 mya) into the Atlantic ocean through the Ogooué valley in Gabon and the Cuanza system in Angola [7], [38]. This indicates that the modern lower Congo rapids cannot be older than 35 mya, and additional analyses of Congo offshore deposits suggest an origin of the present day Congo discharge at the Miocene-Pliocene transition at approximately 5 mya [39], after the southern African continent had been affected by a significant uplift inducing a progressive rearrangement of the watersheds [37]. Our age estimates of cichlid radiations of the lower Congo are fully concordant with this timing, as two phylogenetically independent cichlid lineages radiated within the last 5 mya. Median age estimates for the lower Congo cichlid endemics included here range from 1.6 - 2.67 mya for *Nanochromis* and from 0.94–4.48 mya for *Steatocranus* (Fig. 3). These results corroborate the view that the lower Congo cichlid radiation was closely linked to the establishment of new habitat availability and thus represents an autochthonous radiation within the lower Congo River.

### Biogeographic differentiation along the Lower Congo rapids

Our phylogenetic data correspond to a subdivision of the lower Congo into three biogeographic areas (Fig. 4, see also [40]): (A) upstream (from Malebo Pool to Mbelo, 133 km) (B) central (from Luozi to Kinganga, 129 km) and (C) downstream (from Inga to Boma, 88 km). Each of these river sections is characterized by unique geomorphological settings [40] as well as different species assemblages with various degrees of local endemism not restricted to cichlids ([4], [41] and pers. obs.). The upstream lower Congo (“northern rapids”, [40]) starts with the steep Livingston falls, separating the lower Congo from the Cuvette Centrale, and stretches around 133 km downstream intersected by several smaller rapids. The transition from upstream to the central lower Congo is characterized by a change in sediments from Proterozoic to Precambrian quarzites and schists [5] and the presence of rapids at Mbelo and Bela (Fig. 1). The central lower Congo (ca. 129 km long) is a large navigable tract characterized by a wider lake-like river channel that is occasionally narrow and very deep (up to 200 m around Bulu, [2]). The downstream lower Congo (ca.88 km long, “southern rapids”, [40]) is the steepest river section and mainly characterized by the presence of the huge rapids at Inga and Yalala. A spatial genetic differentiation is apparent in most *Steatocranus* and *Nanochromis* clades whose distribution exceeds one of these river stretches (Fig. 2). The most obvious mechanism shaping the lower Congo species diversity is the complexity of the river itself, with alternating stretches of rapids and deep river habitats [2]. According to our data, the rapids at Inga and Yalala have provided the strongest barriers to dispersal, as supported for example by the elevated degrees of local endemism and ancient splits leading to differences in species composition in both genera. Below Nziya (close to Inga), no *Nanochromis* species occur, and apart from *Steatocranus glaber* only the locally endemic *S*. cf. aff. *tinanti* “intermediate” is present. Downstream of the Yalala rapids only a single *Steatocranus* species (*S. mpozoensis*) is found, even though Matadi (downstream of Yalala) was given as the type locality for *S. gibbiceps* [42]. However, its occurrence could not be verified despite substantial efforts and the type location information likely refers to the port of shipment (“Matadi”) rather than the true collection site. Fine-scale differentiation in two other distantly related lower Congo cichlids [43] matches the above described spatial pattern for the central/downstream lower Congo area. *Lamprologus tigripictilis* forms two well separated populations above and below the Inga rapids and the highly rheophilic cichlid genus *Teleogramma* exhibits a pronounced population structure shaped by smaller rapids (Isangila and Fwamalo) upstream of Inga but is absent below the rapids.

The *S*. cf. *tinanti*, *S*. cf. *gibbiceps*/*S. glaber*/*S. mpozoensis* and *N. splendens*/*N. consortus* species groups diverged roughly 3 mya (3.03, 3.43 and 2.67 mya respectively, Fig. 3) and both lineages evolved locally endemic species in and below the rapids of Inga (e.g. *S. glaber*, *S. mpozoensis, S*. cf. aff. *tinanti* “Inga”, and *N. consortus*). Within the *S*. cf. *tinanti* species complex a phylogenetic split (node age 3.03 mya) separates the Yalala rapids endemic in the downstream part of the lower Congo (*S*. cf. aff. *tinanti* “intermediate”) from the remaining three clades (Fig. 2). At first glance, this basal phylogenetic split between a single downstream and the remaining upstream species appears implausible. In a strongly flowing (continuous) river stretch like the lower Congo a gradual progression and differentiation of populations in flow direction appears more likely. A potential scenario (“large Inga waterfall hypothesis”) explaining this and the high degree of endemism below the Inga rapids is, that following the first colonization wave early colonists of *Steatocranus* and *Nanochromis* have remained strongly isolated in downstream regions since about 3 mya (e.g. by a waterfall at Inga). Riverbed erosion may then have worn down this waterfall resulting in the present day Inga rapids (rather than falls), which are penetrable to upstream movement by fishes.

The formation of the present day lower Congo offered not only new habitat opportunities but also structurally new habitat types, e.g. by the combination of extreme currents and turbidity [4]. Differing spatial structures depending on the species-(group) or genus (Fig. 2) indicate that apart from a prominent role played by extrinsic habitat features of the lower Congo rapids, intrinsic factors shaped the species divergence. Often a synergetic composition of both extrinsic (e.g. habitat composition, physical barriers to gene flow) and intrinsic factors (e.g. dispersal capabilities or ecological adaptations) is responsible for species differentiation [44]. Analogous to the famous “Mbuna” from Lake Malawi [45], [46], the rock-dwelling and strictly rheophilic *Steatocranus* [4] apparently exhibit a higher site fidelity and limited dispersal capabilities compared to the sand-dwelling and less rheophilic *Nanochromis*. Further, intrageneric differences within *Steatocranus* point to alternative ecological adaptations (e.g. differences in dentition and body shape [4]). Most obvious differences are present between the low-bodied *S*. cf. *tinanti* and *S. mpozoensis* and the higher bodied *S*. cf. *gibbiceps* or *S*. cf. *casuarius* [4], indicating differential adaptations to the life in strong current, e.g. on top (*S.* cf. *tinanti, S. mpozoensis*) or among stones (*S*. cf. *gibbiceps*, *S. casuarius*, pers. obs.). However, quantitative ecological data and a denser sampling are needed to differentially analyze causes and factors responsible for the riverine lower Congo species richness. Ecomorphological differentiations in dentition and body shape described for the rock dwelling Eretmodini (genera *Tanganicodus*, *Eretmodus* and *Spathodus*) from Lake Tanganyika correspond to those found in *Steatocranus* [47], [4]. This might indicate either a synapomorphic developmental basis for character evolution at the base of austrotilapiines [18] or a rapid genetically independent origin of trophic adaptations. This highlights the importance of including possible riverine founder species, when analysing the origins of the enormous adaptability of the megadiverse East African cichlid radiations.

### The cichlid species flocks of the lower Congo rapids

The use of the term “species flock” is controversial and has been much debated [19]. Fish species flocks were typically discussed with respect to speciose groups in closed lacustrine environments, of which the cichlid radiations of East African rift valley lakes and Cameroonian crater lakes are the most famous examples [20], [48], [49]. In contrast, riverine species flocks were rarely studied and to date only a few examples exist. Sullivan et al. [50], [51], Feulner et al. [52], [53] and Kullander et al. [54] gave examples for riverine species flocks of weakly electric fish (Mormyridae) in the Ogooué River system in Gabon and the lower Congo River and South American cichlids restricted to the upper Rio Uruguay system, respectively. The distributions of these species flocks were either very broad and encompassed several river systems [50], [51] or intrageneric sampling was not complete with regard to known taxa and sampled areas [52], [53]. To our knowledge, the South American pike cichlids of the *Crenicichla missioneira* species group currently represent the best candidate for a riverine species flock, even though the phylogenetic study [54] was based on only a single mitochondrial marker and the taxon sampling did not contain all important members. Here, we present the first evidence for a riverine species flock and several species pairs endemic to an exceptionally complex habitat.

The *S*. cf. *tinanti*/ *S*. cf. *gibbiceps*/ *S. glaber* and *S. mpozoensis* species group is endemic to the lower Congo rapids, a defined geographic area, and forms closely related assemblages, thereby meeting the three species flock criteria. *Nanochromis splendens* and *N. consortus* and *S*. *casuarius* and the yet undescribed species *S*. cf. aff. *casuarius* “brown pearl” each form species pairs endemic to the lower Congo rapids. Both genera colonized the lower Congo rapids in at least two allochronic events each forming independent closely related riverine cichlid species assemblages (Fig. 2) within a timeframe of 5 mya.

### Conclusion

The rapids of the lower Congo River, inhabited by a remarkable diversity of cichlids and other fishes, provide an outstanding example for the underestimated diversity of riverine and especially rapids systems. Like lakes and islands, rapids provide novel ecological opportunities for riverine organisms and often form after catastrophic geomorphological events. Due to steep selectional gradients between average riverine conditions and rapids habitats, invading species facing multiple unutilized ecological opportunities may rapidly adapt to these extreme conditions and form locally endemic species assemblages. Our data suggest that multiple (minimum two) allochronic colonization events seeded the present day diversity of the lower Congo *Steatocranus* and *Nanochromis* species, which subsequently evolved into small species flocks. Cichlid species diversity in the lower Congo is arranged sequentially and differentiated ecologically. This study provides a phylogenetic primer for the study of this complex system, that may serve as a link between riverine diversity and the megadiverse cichlid radiations of the East African lakes, which - based on the inherent logic of lakes being interconnected only by rivers - surely were seeded by riverine cichlids [10]. In particular the close phylogenetic relationship of the *Steatocranus* species to the East African cichlid radiations [18] renders this system appealing.

## Supporting Information

Figure S1 **Branch attachment frequency.** Alternative positions of the unstable Southern clade in 1000 bootstrap topologies. The numbers, plotted on the neighbour joining tree (based on the AFLP dataset), indicate fractions of bootstrap trees in which alternative branching patterns occur.
(TIF)

Table S1 **Specimen information.** Overview of samples used for the mt- and AFLP dataset.
(XLS)

Table S2 **Overview of samples and genes used for molecular clock dataset.**
(XLS)
