# Supplementary figures and images for: Time and Origin of Cichlid Colonization of the Lower Congo Rapids
Source: PLoS One. 2011 Jul 20;6(7):e22380. doi: 10.1371/journal.pone.0022380 (PMC3140524; doi:10.1371/journal.pone.0022380)

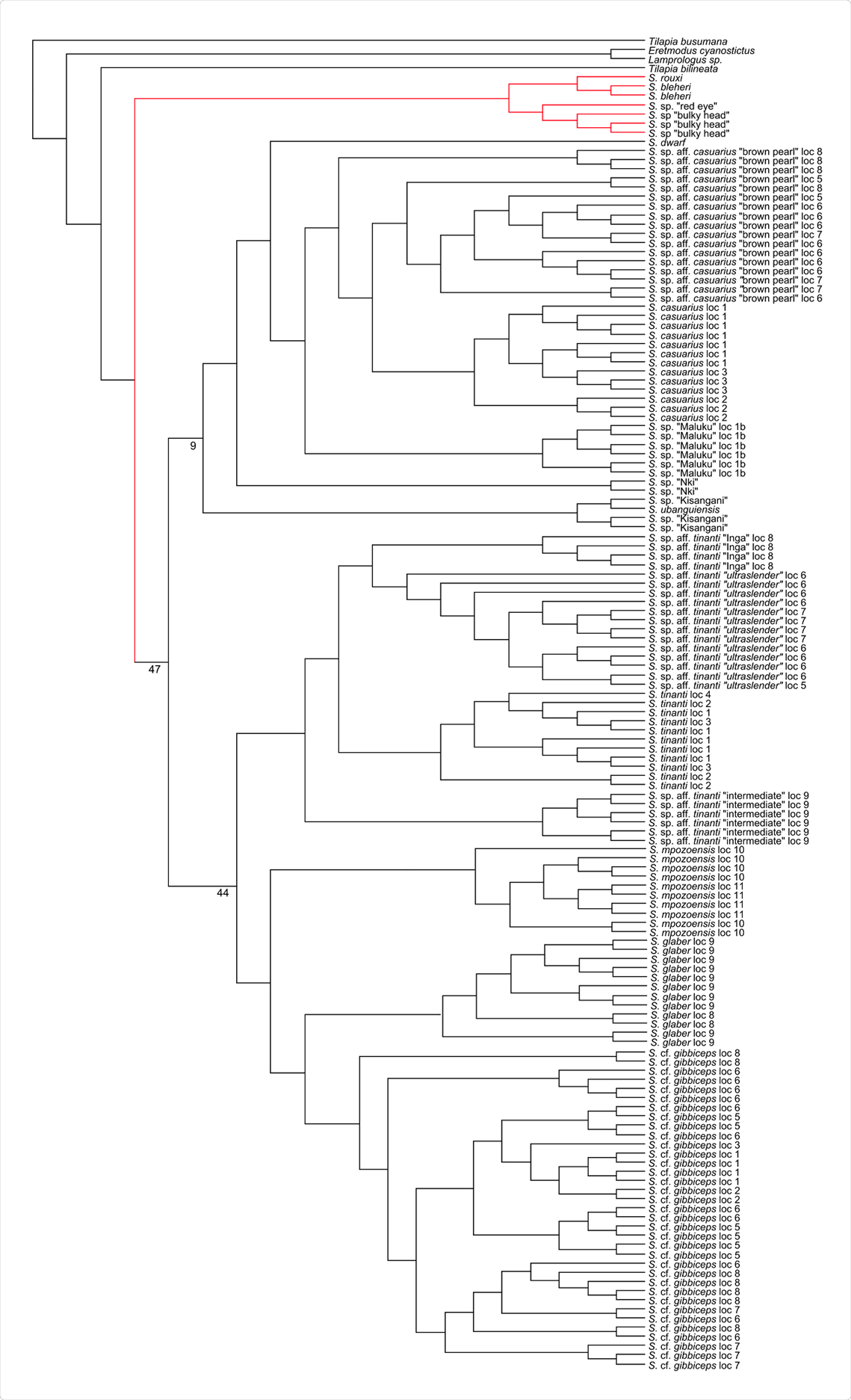

Supplement: Figure S1 — Branch attachment frequency. Alternative positions of the unstable Southern clade in 1000 bootstrap topologies. The numbers, plotted on the neighbour joining tree (based on the AFLP dataset), indicate fractions of bootstrap trees in which alternative branching patterns occur. (TIF) [file pone.0022380.s001.tif]
